# Feature Extraction of Shoulder Joint’s Voluntary Flexion-Extension Movement Based on Electroencephalography Signals for Power Assistance

**DOI:** 10.3390/bioengineering6010002

**Published:** 2018-12-24

**Authors:** Hongbo Liang, Chi Zhu, Yu Iwata, Shota Maedono, Mika Mochita, Chang Liu, Naoya Ueda, Peirang Li, Haoyong Yu, Yuling Yan, Feng Duan

**Affiliations:** 1 Department of Environment and Life Engineering, Graduate School of Engineering, Maebashi Institute of Technology, 460-1 Kamisadori, Maebashi, Gunma 371-0816, Japan; m1756503@maebashi-it.ac.jp; 2 Department of Environment and Life Engineering and Department of Systems Life Engineering, Maebashi Institute of Technology, 460-1 Kamisadori, Maebashi, Gunma 371-0816, Japan; zhu@maebashi-it.ac.jp; 3 Department of Systems Life Engineering, Graduate School of Engineering, Maebashi Institute of Technology, 460-1 Kamisadori, Maebashi, Gunma 371-0816, Japan; m1756002@maebashi-it.ac.jp (Y.I.); m1856012@maebashi-it.ac.jp (S.M.); m1656001@maebashi-it.ac.jp (N.U.); m1856015@maebashi-it.ac.jp (P.L.); 4 Department of Systems Life Engineering, Faculty of Engineering, Maebashi Institute of Technology, 460-1 Kamisadori, Maebashi, Gunma 371-0816, Japan; m1571044@maebashi-it.ac.jp; 5 Department of Bioengineering, Faculty of Engineering, National University of Singapore, Singapore 119077, Singapore; bieyhy@nus.edu.sg; 6 Department of Bioengineering, School of Engineering, Santa Clara University, Santa Clara, CA 1304, USA; yyan1@scu.edu; 7 Department of Automation, College of Information Technical Science, Nankai University, Tianjin 300071, China; duanf@nankai.edu.cn

**Keywords:** brain-machine interface, power assistance system, feature extraction, shoulder joint

## Abstract

Brain-Machine Interface (BMI) has been considered as an effective way to help and support both the disabled rehabilitation and healthy individuals’ daily lives to use their brain activity information instead of their bodies. In order to reduce costs and control exoskeleton robots better, we aim to estimate the necessary torque information for a subject from his/her electroencephalography (EEG) signals when using an exoskeleton robot to perform the power assistance of the upper limb without using external torque sensors nor electromyography (EMG) sensors. In this paper, we focus on extracting the motion-relevant EEG signals’ features of the shoulder joint, which is the most complex joint in the human’s body, to construct a power assistance system using wearable upper limb exoskeleton robots with BMI technology. We extract the characteristic EEG signals when the shoulder joint is doing flexion and extension movement freely which are the main motions of the shoulder joint needed to be assisted. Independent component analysis (ICA) is used to extract the source information of neural components, and then the average method is used to extract the characteristic signals that are fundamental to achieve the control. The proposed approach has been experimentally verified. The results show that EEG signals begin to increase at 300–400 ms before the motion and then decrease at the beginning of the generation of EMG signals, and the peaks appear at about one second after the motion. At the same time, we also confirmed the relationship between the change of EMG signals and the EEG signals on the time dimension, and these results also provide a theoretical basis for the delay parameter in the linear model which will be used to estimate the necessary torque information in future. Our results suggest that the estimation of torque information based on EEG signals is feasible, and demonstrate the potential of using EEG signals via the control of brain-machine interface to support human activities continuously.

## 1. Introduction

In recent years, in advanced countries, especially in Japan, the rapid population aging leads to an increasing number of people who cannot move on their own ability. Meanwhile, the caregiving issue is also becoming more serious, because the caregivers’ burden will continuously increase in the future. As an approach to mitigate or solve these problems, various power assistance devices have been developed around the world [1,2,3,4,5,6,7].

In the past, electromyography (EMG) signal, a human biological signals, is widely used to control the power assistance devices [8]. However, EMG signal on the paralyzed side of a patient is weak, sometimes it is even impossible to be generated when the motor nerves are damaged. In such cases, the power assistance devices based on EMG signal are not suitable for those people. On the other hand, as an effective communication method for such people with physical disabilities, Brain-Machine Interface (BMI) is developed to solve those problems by only using the brain activity information [9,10,11].

Because of the clear/distinguishable temporal resolution on electroencephalography (EEG) signals, real-time control of external equipment has been realized (e.g., the BMI-wheelchair, keyboards, etc.). In recent years, studies on estimation and reconstruction of motion information, such as 3-dimensional motion speed of the hand and muscle electromyography from brain activities by a learning-based model have been conducted [12]. A. Y. Peak et. al. developed a neural decoder that reconstructed the surface electromyogram to control the robot gripper by brain waves [13]. By these methods, it is possible to control the robot by brain activities. However, the EEG signals in these studies are just used as a trigger for the switch control. Several motion patterns for robots have to be prepared in advance, then select one of the patterns to realize the control by recognizing the motion feature extracted from the EEG signal.

On the other hand, a power assistance system requires continuous control signals to realize the real-time control. Conventionally, it is difficult to convert an EEG signal to a continuous electrical signal because the shoulder joint is the most flexible joint in a human being. The control of the exoskeleton power assistance device of the upper limb is, thus, more complicated than the control of other joints such as elbow joint. Up so far, the control of a wearable exoskeleton to support human’s shoulder joint by EEG signals has not yet been reported.

In our previous study, we developed a power assistance system controlled by EMG signals that aims at supporting the elderly or physically handicapped [14]. Because of the linear relationship between the EMG signals and the torque [15], if we can clarify the relationship between the EMG signals and the EEG signals, it is possible to design a power assistance system that can be controlled by the torque estimated from the EEG signals. In this research, we aim at estimating the minimum necessary torque information for controlling an exoskeleton robot to perform the power assistance of the upper limb without using any external torque sensors or EMG sensors. This is an unexplored area so far. In this way, not only the healthy people can definitely control the power assistance system, but also the disabled who cannot generate EMG signal or are lack of motion functions can also control it by their own intention directly.

Since there is no report about how to control the shoulder joint of a multi-joint power assistance exoskeleton robot by EEG signals, as the first step, we extract the motion-relevant EEG signals’ features of the shoulder joint for conducting flexion and extension movement freely which are the main motion of the shoulder joint needed to be assisted. In this paper, Independent component analysis (ICA) is used to extract the source information of neural components firstly, and then the averaging method is used to extract the characteristic signals that are fundamental to achieve the control. The proposed approach has been verified by experiments of conducting the flexion and extension movement of shoulder joint. The results show that EEG signals begin to increase at 300–400 ms before the motion and then decrease at the beginning of the generation of EMG signals, and the peaks appear at about one second after the motion. At the same time, we also confirmed the relationship between the change of EMG signals and the EEG signals on the time dimension, and these results also provide a theoretical basis for the delay parameter in the linear model which will be used to estimate the necessary torque information in future. Our results suggest that the estimation of torque information based on EEG signals is feasible, and demonstrate the potential of using EEG signals via the control of brain-machine interface to support human activities continuously.

## 2. Method

### 2.1. Feature Extraction of the EEG Signals Related to the Motion of the Shoulder Joint

In order to find out the EEG information that is related to the motion of the shoulder joint, it is necessary to extract the source signals from the recorded scalp channel data. It is considered that the recorded one channel scalp data is the voltage difference between the source signal projections and the reference channel after propagation and attenuation. Therefore, the so-called EEG signal measured on each electrode is actually a manifestation of a combination of various signals via volume conduction, including various non-cortical source signals (e.g., potentials induced by eyeball movements or produced by single muscle activity, etc.) and other non-physiological signals (e.g., linear noise, equipment noise, environment noise, etc.). The volume conduction process is passive, linear, and adds no extra information to the data [16]. However, it also mixes and obscures the functionally distinct and independent source contributions. Here we take ICA to separate the source signals from the noises. ICA is a widely used method for separating mixed signals and extracting specific signal components in signal processing, especially for EEG signals [17]. It is a multi-dimensional signal analysis method that seeks transformation to separate signals by higher order statistics or independence based on temporal correlation. It separates the signal only based on the assumption that the source signal sources are statistically independent. For this reason, it is also called as Blind Source Separation (BSS). If our measured signals (the source signals related to the motion, non-cortical source signals, and other non-physiological signals) are independent, ICA’s assumption lends itself well to the measured signals, and the feature extraction problem from EEG signals is equivalent to the BSS problem. Thus, in this paper, ICA is applied on the acquired EEG data to extract the components related to the motion of the shoulder joint.

We suppose the signal source s(t) is given by the following vector:

(1) $$s\left(t\right)={\left(s_{1}\left(t\right),s_{2}\left(t\right),\ldots ,s_{n}\left(t\right)\right)}^{T}\;\;\;t=0,1,2,\ldots$$

The mean of each signal source of $s\left(t\right)$ is 0, and each signal source is independent from each other. ${\left(\cdot \right)}^{T}$ represents transpose operation. The measured signal is denoted as follows:

(2) $$x\left(t\right)={\left(x_{1}\left(t\right),x_{2}\left(t\right),\ldots ,x_{m}\left(t\right)\right)}^{T}\;\;\;t=0,1,2,\ldots$$

These signals can be considered as the signals observed with m sensors. Here, we assume a linear relationship between $s\left(t\right)$ and $x\left(t\right)$ as follows.

(3) $$x\left(t\right)=As\left(t\right)$$

A is an m×n real matrix. The problem of BSS is to separate $x\left(t\right)$ into n independent signal components without any prior information about the probability distribution of $s\left(t\right)$ and A. If n≤m, The solution exists, that is, there is a certain n×m real matrix W to reconstruct mutually independent $y\left(t\right)$ defined as follows:

(4) $$y\left(t\right)=Wx\left(t\right).$$

If WA=I (I is an identity matrix of n×n size), $s\left(t\right)$ equals $y\left(t\right)$. So in the ICA method, the independent component filters are chosen to produce the maximally temporal independent signals $s\left(t\right)$ that are available in the observation data $x\left(t\right)$. In this paper, the method is called the logistic infomax ICA algorithm that maximizes the amount of information is used to perform the decomposition [18], and the EEG signals are processed in MATLAB environment.

### 2.2. Averaging Method

The averaging method is a process that can reduce noise components and extract characteristic signal components at the same time by obtaining the average of the same measurements multiple times [19]. In EEG signals, in order to extract the changes of potential due to certain stimuli, the averaging method is used to eliminate the background EEG signals that are always generated and mixed in every measurement as a noise.

When m-times averaging is performed, starting point in each measurement is aligned. The waveform x(k) after averaging is calculated as:

(5) $$\begin{array}{ccc} x(k) & = & \frac{1}{m}\sum_{i=1}^{m}x_{i}\left(k\right)\;\left(k=0,1,2,\ldots \right),\;\left(i=1,2,3,\ldots \right), \end{array}$$

where *i* represents each measurement data, and *k* represents the sample number. For example, $x_{i}\left(k\right)$ represents the *k*-th sample number in the *i*-th measurement data.

After the averaging method, the average value of the signal components is the original amplitude. However, the noise components are eliminated statistically. In general, the probability distribution of noise can be approximated as a normal distribution of mean $\mu_{n}$, and variance $\sigma_{n}^{2}$, and the amplitude of the noise component is usually defined as the square root of the variance. That is to say, the amplitude of the noise component after the averaging becomes $1/\sqrt{M}$. Table 1 summarizes the number of averaging times and the improvement of the signal-noise ratio(SNR). Since the number of addition average in this experiment is between 60 and 80, the noise component can be suppressed to approximately one-ninth to one-eighth.

## 3. Measurement

### 3.1. Experimental Setup

**Figure 1:**
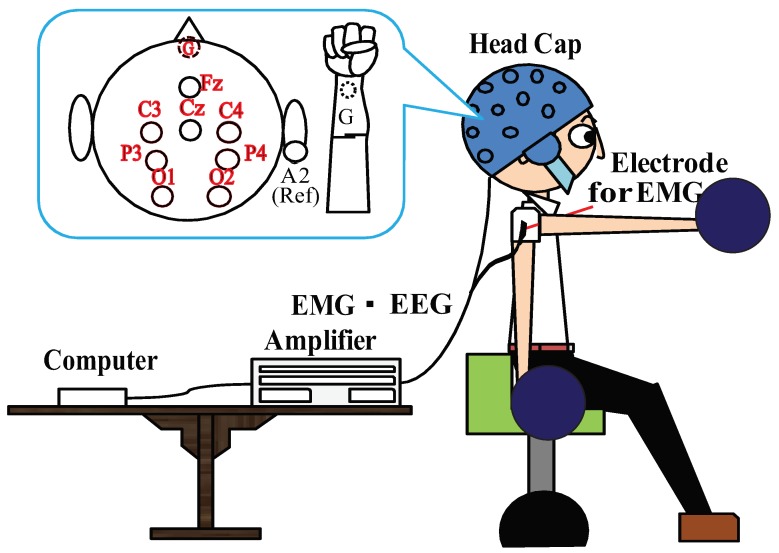
Measurement of EEG and EMG signals. A participant in the experimental trial is sitting on a chair with the eyes open and performing flexion and extension of the shoulder joint with a weight of 3 Kg. The movement is conducted for a duration about 60–80 times, consecutively within 5 min with their own pace. He wears an EEG measurement cap, the electrodes fixed on the cap and an electrode for the measurement of EMG signal are connected to the EEG amplifier and EMG amplifier, respectively. The amplified data are transmitted to the computer after the analog/digital(A/D) conversion and recorded.

In this experiment, the subjects were three healthy right-handed men in their 20’s who were well informed of the procedure and consented in advance. The research has an approved IRB (Institutional Review Board) protocol by Maebashi Institute of Technology. g.GAMMAsys (g.tec. Co.) and g.BSamp (g.tec. Co.) were used for EEG signal recording. Active electrodes mounted with the g.GAMMAcap onto the head were used to reduce artifacts from movements and electromagnetic interference. The measurements of EMG signal were collected by DELSYS’s electrodes and amplifiers. In our experiments, the EMG signals were just collected for defining the beginning time of the motion and the latency time related to the change of the EEG source signals in the initial stage. In the future applications, the measurement of the EMG signals is not required, as a purpose of this study, for healthy people and the disabled. The interface board (HRP Interface Board 07-0003-1, ZUCO. Co.) was used to synchronize EEG signals with EMG signals. The sampling frequency of 1000 Hz is used for both EEG and EMG data acquisitions. The whole experimental setup is shown in Figure 1.

### 3.2. Experimental Task

As shown in Figure 1, subjects sit on a chair with the eyes open and perform flexion and extension of the shoulder joint with a weight of 3 Kg. The movement is conducted for about 60–80 times consecutively within 5 min with their own pace. Considering the real-time measurement and control of the system, it is necessary to reduce the number of electrodes and signal processing burden. In this study, based on the International 10-10 system we select 8 points as: Fz, C3, C4, Cz, P3, P4, O1 and O2, respectively [20,21,22,23,24]. The reference of the EEG electrodes is set at their right earlobe. The ground of the EEG electrodes is set at FpZ. The differential amplification is carried out between the earlobe and 8 measurement points, respectively. In order to detect the beginning time of the motion, EMG signal on the acromial deltoids muscle of the right shoulder is measured and recorded simultaneously with EEG signals measurement. The right wrist is taken as the body reference.

### 3.3. EEG and EMG Signal Processing

#### 3.3.1. EMG Signal Processing

Because there is no fixed time reference for voluntary movement, here we use the change time of the amplitude of the EMG signals to represent the beginning time of the voluntary movement. The procedure for the EMG signal processing is briefly described as follows. The EMG signal after A/D conversion is first processed by a full-wave rectifier. Then the signal is smoothed with a 20-point moving average, and followed by a Butterworth low-pass filter (cut off frequency of 0.7 Hz). An appreciable decrease in the amplitude is observed in the EMG signal after the smoothing and filtering in comparison with the original signal. To restore the original amplitude, we set a recovery factor of 4. In order to eliminate individual differences, the smoothed data matrix is processed by normalizing each data set’s minimum and maximum values to [0, 1] as follow:

(6) $$y=\frac{x-x_{min}}{x_{max}-x_{min}}$$

where $x_{min}$ and $x_{max}$ represent the minimum and maximum values of each EMG signal data set, respectively.

**Figure 2:**
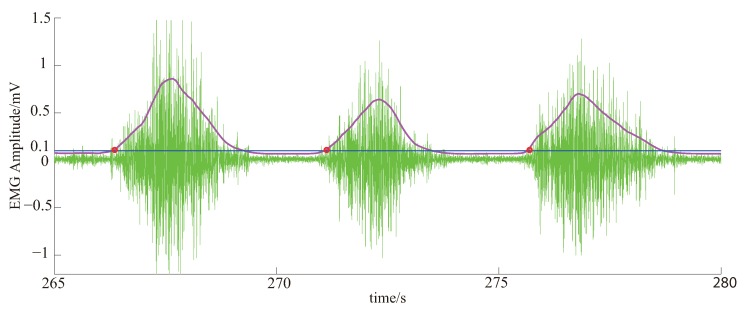
Definition of the time when the motion starts. The green waves are the raw EMG signals, the red line is smoothed and normalized EMG signals after adjusted for the filter delay, the blue line is the positions where the normalized EMG value is 0.1, and the red circles are the time when the motion starts.

Due to the use of moving average and low pass filters, there is a certain delay between the filtered waveform and the original data, which is approximately 250 milliseconds. As a kind of control signal, EMG can be regarded as a transitional response signal, here 10% of the curve maximum is considered as the starting point. Because the EMG signal is normalized, 10% of the curve maximum is the value of 0.1 in the EMG envelope. Therefore, the time before 250 milliseconds of the value of 0.1 in the EMG envelope is set as the time when the motion starts as shown in Figure 2. It has been empirically confirmed that the extracted time aligns with the actual time of motion beginning.

#### 3.3.2. EEG Signal Processing

Following A/D conversion, the EEG signals are processed by a Butterworth high-pass filter (cut-off frequency of 1 Hz) to remove the baseline drift. Then, the EEG signals are processed by ICA to obtain the independent components. As mentioned above, assuming the time at the 10% of EMG signal’s amplitude is 0 s for data analysis, a total of 3 s from 1 s before motion start to 2 s after motion are cut out as one epoch from every independent component. In order to reduce the effect of baseline differences between data epochs (e.g., those arising from low-frequency drifts or artifacts) and avoid corrupting the data analysis, a mean baseline value from each epoch is removed. At last, the averaging process of the total number of trials in each operation is applied to the EEG data, and the trend of the change is investigated in the following section.

## 4. Results

### 4.1. Results and Discussion of ICA and Components Presumption

**Figure 3:**
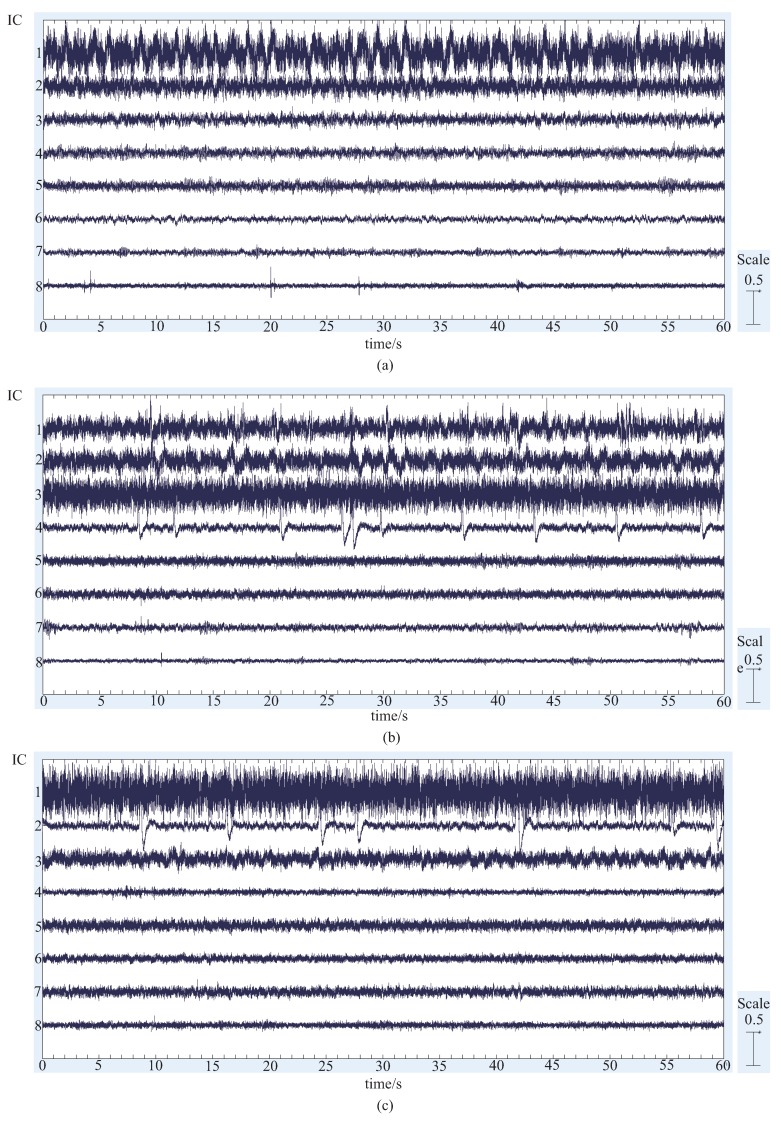
Independent component analysis results of three subjects (**a**–**c**) for one-minute data. The horizontal axis is the time in seconds, and the vertical axis is the amplitude of the independent component. The numbers from 1 to 8 correspond to 8 independent components.

First, the results of the ICA of the three subjects are shown in Figure 3.

Since eight EEG signal channels were measured, we can decompose the data up to 8 meaningful independent components at most. However, we are not sure how many components are included in the measured EEG data, we will confirm all the 8 components obtained by ICA, and extract the characteristic components. Since the data is too long to see the details, only one-minute data is shown in the figures. In the figures, the horizontal axis is the time in seconds, and the vertical axis is the amplitude of the corresponding component. The numbers from 1 to 8 correspond to 8 independent components. From the waveforms of the ICA results of the three subjects, we can roughly classify these components into the following categories:

1. The similar components are considered as the most likely motion-related component, in other words, a characteristic component of the EEG signals during the motion performing.
2. Intermittent pulse components, which are considered as the noise introduced by eye-blinking or eye movement.
3. The components with small amplitudes and no obvious changes, which are considered as the background EEG signals.
4. And other tiny noise components.

**Figure 4:**
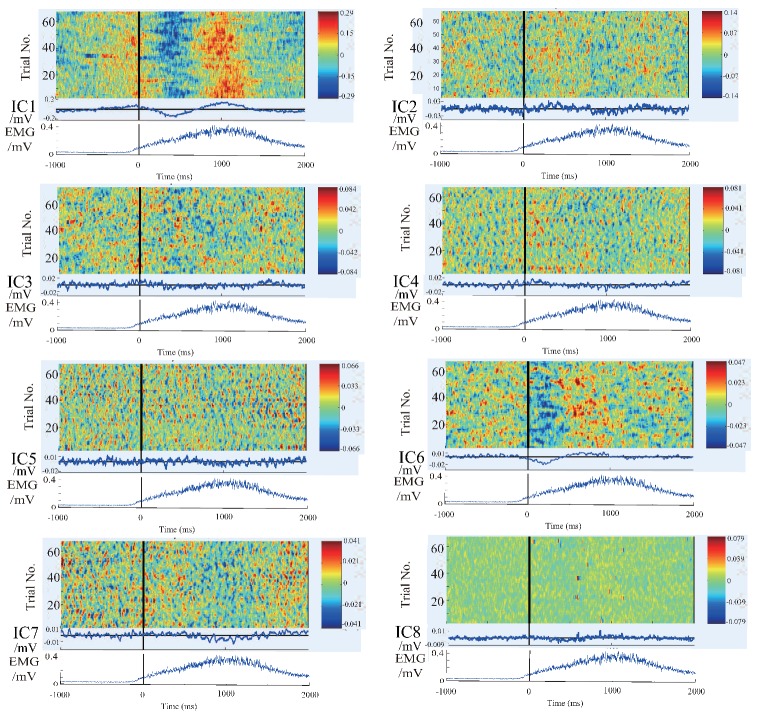
The waveform of each independent component in each experiment of subject A. 8 subpanels in the figure correspond to 8 independent components (IC1–IC8). The upper part of each subpanel shows the amplitude of the independent component restored to all trials at time series, which is plotted by color. The horizontal axis is time, and the black vertical line divides the 3-s data to 1 s before the motion and 2 s after the motion. The vertical axis is the number of trials. The IC part is an average value of EEG signals of all the waveforms produced by all the trials. The lower part is an average value of EMG signal of all the trials.

**Figure 5:**
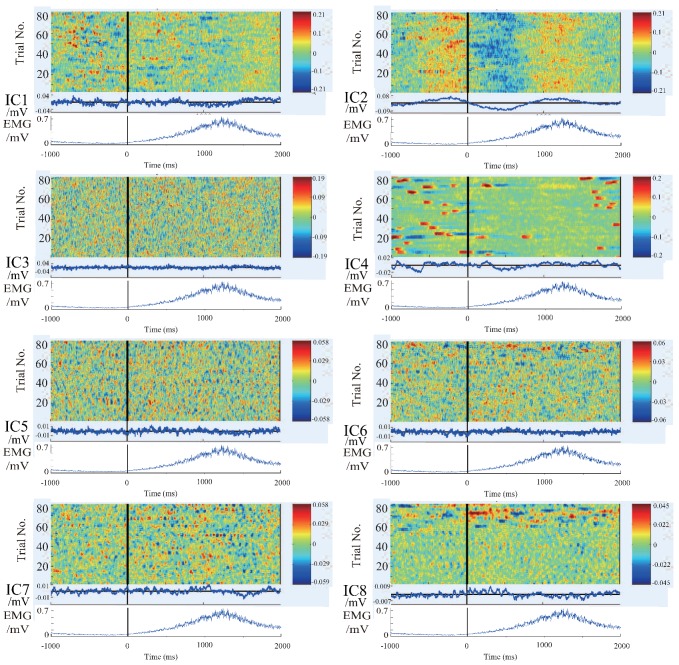
The waveform of each independent component in each experiment of subject B. 8 subpanels in the figure correspond to 8 independent components (IC1–IC8). The upper part of each subpanel shows the amplitude of the independent component restored to all trials at time series, which is plotted by color. The horizontal axis is time, and the black vertical line divides the 3-s data to 1 s before the motion and 2 s after the motion. The vertical axis is the number of trials. The IC part is an average value of EEG signals of all the waveforms produced by all the trials. The lower part is an average value of EMG signal of all the trials.

**Figure 6:**
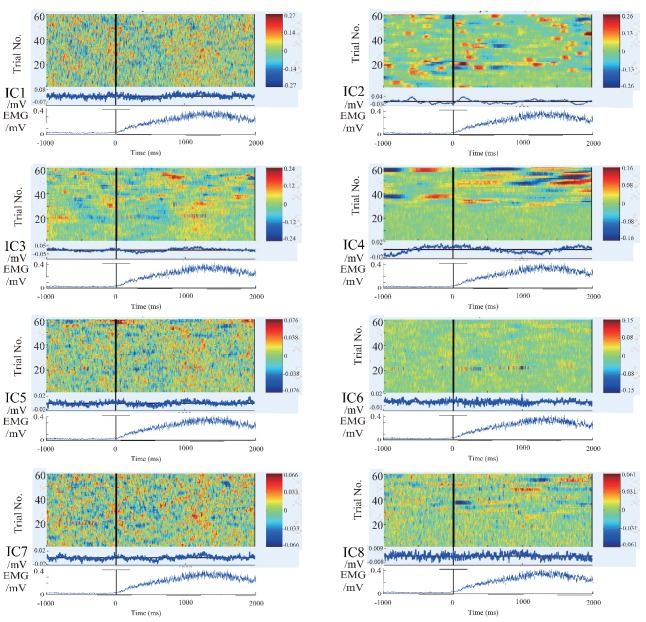
The waveform of each independent component in each experiment of subject C. 8 subpanels in the figure correspond to 8 independent components (IC1–IC8). The upper part of each subpanel shows the amplitude of the independent component restored to all trials at time series, which is plotted by color. The horizontal axis is time, and the black vertical line divides the 3-s data to 1 s before the motion and 2 s after the motion. The vertical axis is the number of trials. The IC part is an average value of EEG signals of all the waveforms produced by all the trials. The lower part is an average value of EMG signal of all the trials.

In order to further clarify the actual physical meaning of each component, the independent component signal is restored to each measurement data, and the waveform of each independent component in each trial is plotted to figure out the feature of the independent component. The restored results are shown in the Figure 4, Figure 5 and Figure 6.

**Figure 7:**
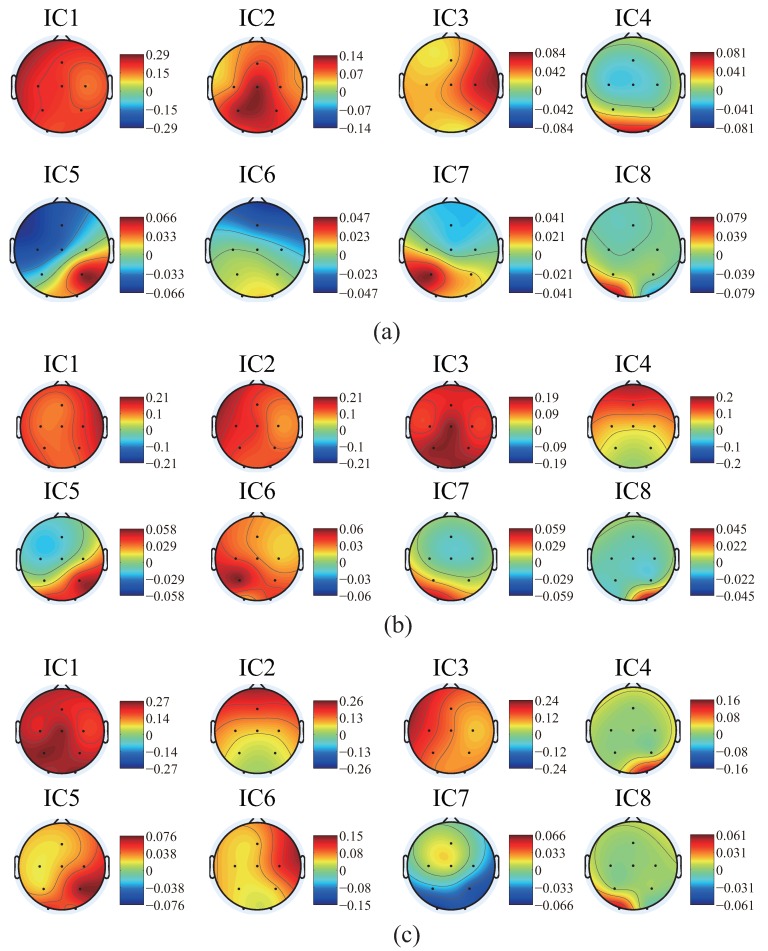
The average values of each independent component (IC) on the scalp of subject A (**a**), B (**b**) and C (**c**) based on the data in Figure 4, Figure 5 and Figure 6.

There are 8 subpanels in each figure, which correspond to 8 independent components. The upper part of each subpanel is the waveform of the independent component restored to all trials in the experiments. The horizontal axis is time, and the black vertical line divides the 3-s data to 1 s before the motion and 2 s after the motion. The vertical axis is the number of trials. The IC part is an average value of EEG signals of all the waveforms produced by all the trials. The lower part is an average value of EMG signal of all the trials. In order to know the locations of the EEG signals’ changes more intuitively, the averaged waveform distribute on the scalp is plotted in Figure 7 based on the data in Figure 4, Figure 5 and Figure 6.

We summarize the results in Figure 4, Figure 5, Figure 6 and Figure 7 to define the components through the index of amplitude, variation, and location, and get some interesting conclusions. First, through these four pictures, we can basically figure out the physical meaning of each independent component. Therefore we can identify the component related to the motion. From Figure 7, we can confirm a similar component in all the subjects, the first independent component (IC1) of subject A, the second IC (IC2) of subject B, the third IC (IC3) of subject C. Moreover, the trend of these three components in the three maps is also almost the same, that is, the EEG signals gradually increase before motion starts, and immediately weaken at the moment of motion start, then increase again after motion starts, and finally resume. The changing moment only depends on the time of motion start, and every time the change is basically the same, so this component is considered as the EEG characteristic signal directly related to the motion.

**Figure 8:**
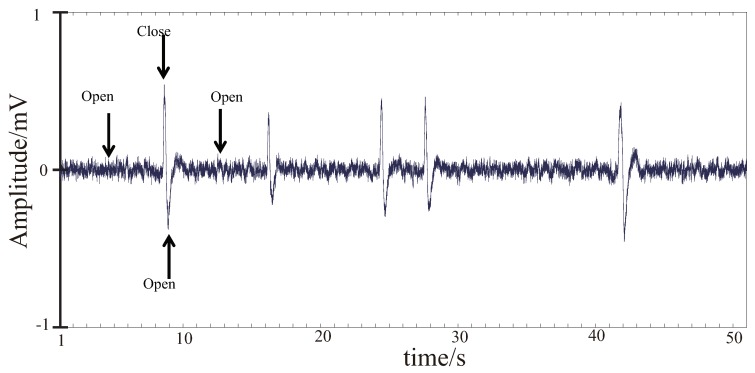
The characteristic waveform of the potential change caused by eye-blinking decomposed from measured EEG signals by ICA. By comparing with the video recorded during the experiments, the results show that the potential does not change substantially when the eyes open. At the time of eyes closed, a positive potential is generated, and when the eye opened again, a negative potential is generated. After the blink, the potential returns to 0 again.

**Figure 9:**
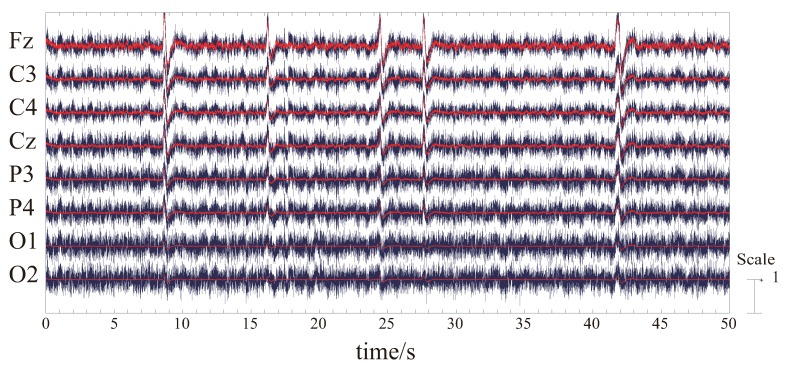
The relationship and influence between eye-blink component and EEG signals. The blue line is measured EEG signals, and the red line is the eye-blink component of Figure 8.

Second, we can clarify the components related to the movement of the eye or eye-blinking. From the result, we know that this component in subject A is not obvious since the subject A tried his best to control the eye-blink when conducting the motion. Conversely, in the data of subject B and subject C, we can clearly observe much single violent potential vibrations. Furthermore, the locations of these changes are also distributed in the area near the eyes. For these reasons, we compared the time between the eye-blinking and the potential change through the video recorded during the experiments, and confirm that the change time between the two is completely consistent. From these results, we can grasp the characteristics of the eye-blinking component and the location of the distribution. The characteristic waveform of the potential change caused by eye-blinking is shown in Figure 8, and the relationship and influence between eye-blink component and EEG signals is shown in Figure 9 as follows.

Third, some signals corresponding to visual feedback, or background EEG has been found. There are some components, which are concentrated in the visual area such as O1, O2. These signals may be the visual feedback signal (e.g., pay attention to the position of the hand, observe the movement of the hand, etc.), or the background EEG signals caused by external stimulation in daily life (other sensory stimuli). Since the amplitude of the change of these signals is relatively small and not stable, in this paper, we will not do a further detailed analysis. These signals will be compared and confirmed after visual feedback tasks are imported in the future work.

### 4.2. Results and Discussion of Feature’S Average

**Figure 10:**
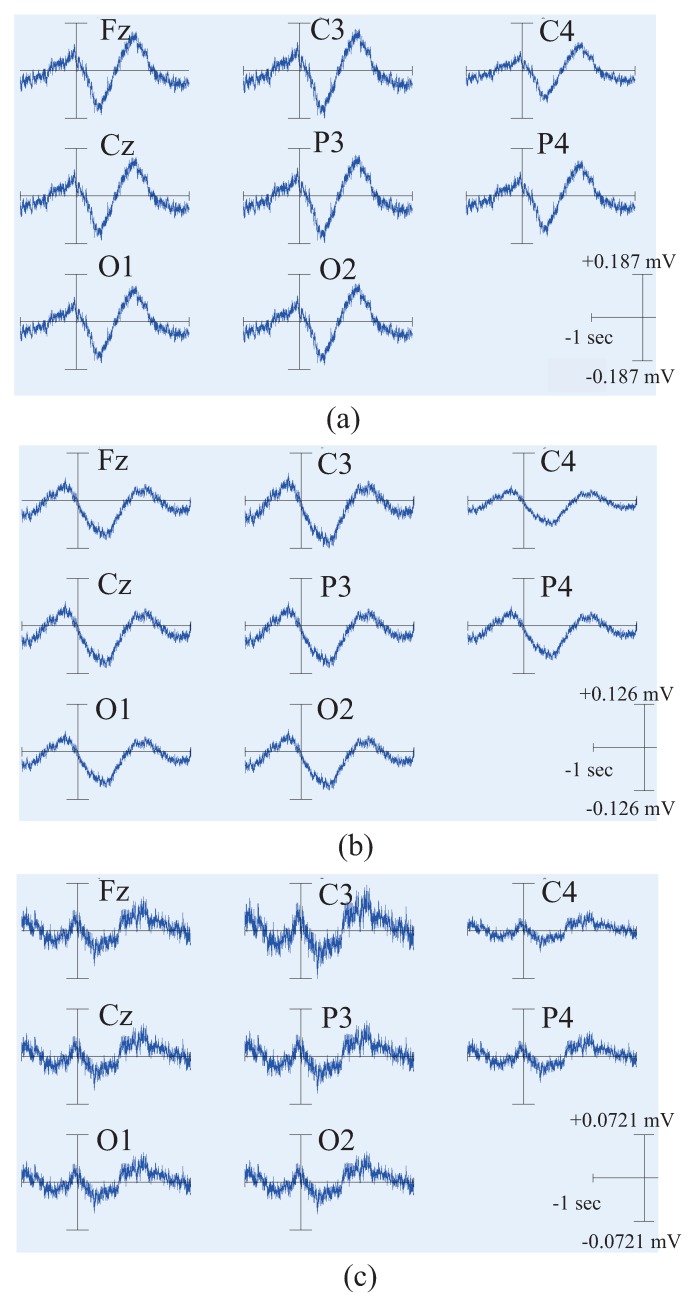
Average results of the feature component distributed in each channel of subject A (**a**), B (**b**) and C (**c**). Each subpanel shows the result of each measurement channel. Here, the horizontal axis is time and the vertical axis is the amplitude of EEG signals.

As mentioned above, we have distinguished the EEG components directly related to motion. Then we extracted the average signals of this component, and the number of trials is 68, 80, and 61 for subject A, B, and C, respectively. Since it is a voluntary movement, the number of actual movements for each subject is different. The results are shown in Figure 10.

It can be seen that the trend and location distribution of this component of the three subjects are relatively stable and universal. Moreover, this change of EEG signals is not only found on one of the electrodes, but actually observed in all the channels. That is to say, when performing the motion, the brain does not work only with specific brain region, but rather works together, reflecting the characteristics of the brain collaboration.The results also show that the amplitude on the left hemisphere is significantly larger than the one on the right hemisphere. This is caused by the fact that the three subjects in this research are all right-handed, and all perform the experiments with their right hands. The lateral phenomenon for flexion and extension of the shoulder exists. Furthermore, subject A and B are good at sports, causing not too much high-frequency waveform in the components. Conversely, the high-frequency waveform is more prominent in the case of subject C, who acquires low-motor-ability.

### 4.3. Results and Discussion of the Relationship Between EEG Signal and EMG Signal

**Figure 11:**
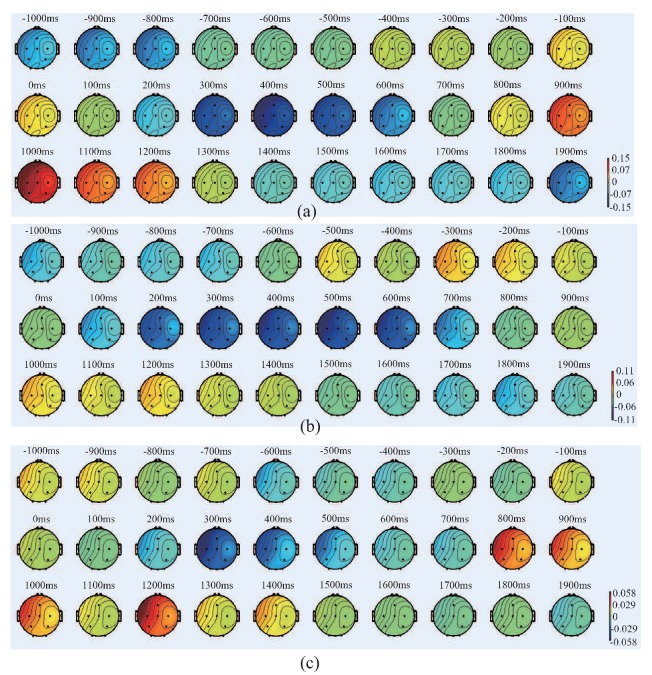
Timing diagram of the averaged EEG signals. In the (**a**–**c**) represents the results of three subjects, respectively. The data of 3 s for each subject is divided into three rows, corresponding to 1 s before the motion, 1 s after the start of the motion and 1 s to 2 s after the motion starts. The colors in the figure correspond to the amplitude of processed EEG signals.

In order to clarify the specific moments of changes of EEG signals, we plot the timing diagram of the averaged EEG signals, as shown in Figure 11, in which (a), (b), (c) represents the results of three subjects.

The data of 3 s for each subject is divided into three rows, corresponding to 1 s before the motion, 1 s after the start of the motion and 1 s to 2 s after the motion starts. The colors in the figure correspond to the amplitude of processed EEG signals. The figure of (a) shows that the EEG signals begin to increase at 400 ms before the motion start, and then decay until the beginning of the motion. After that, it reaches the maximum amplitude in the negative direction at 400 ms after the movement, and then slowly increases and reaches the maximum amplitude in the positive direction at 1 s after the motion. In the same way, subject B’s EEG signals start to increase at 300 ms before the motion and then decay until the beginning of the motion. It reaches the maximum amplitude in the negative direction at 500 ms after the motion, and then slowly increases to the maximum amplitude in the positive direction at about 1.1 s after the motion.

Subject C’s EEG signals start to increase at 300 ms before the motion, and decay until the beginning of the motion. And then it reaches the maximum amplitude in the negative direction at 300 ms after the movement, and then slowly increases and reaches the maximum amplitude in the positive direction at 1.2 s after the movement.

Thus, it seems that the feature signals start to increase at 300–400 ms before the motion and decay until the start of the motion. And then reach the maximum amplitude in the negative direction at 300–500 ms after the motion, and increase slowly and reach the maximum amplitude in the positive direction at 1–1.2 s after the motion. From these results, we clearly understand that EEG signals have begun to change at 300–400 ms before the generation of EMG signals. Based on the aforementioned change patterns, the changes in EEG signals are not a single change by a single pulse signal, but a continuous change. We speculate that the changes in the initial phase are likely related to the motion plan, and changes in the later period are related to the magnitude of the output torque. Further research will be conducted in the future.

## 5. Conclusions

In this research, we aim to realize BMI that can reflect human motion intentions in the power assiatance devices. A feature extraction method is proposed, and the characteristic EEG signals are effectively extracted when the shoulder joint is doing flexion and extension which is the main motion of the shoulder joint needed to be assisted. ICA is used to extract the source information of neural components, and then the average method is used to extract the characteristic signals that are beneficial to realize the control. As a result, one of the components is extracted and considered as the component related to the motion of flexion and extension of shoulder joint. The results show that EEG signals begin to increase at 300–400 ms before the motion and then decrease at the beginning of the generation of the EMG signals, i.e, decrease at the beginning of the actual motion, and the peaks appear at about one second after the motion. At the same time, we also confirmed the relationship between the change of the EMG signals and the EEG signals on the time dimension, and these results also provided a theoretical basis for the delay parameters in the linear model which will be used to estimate the necessary torque information in future. In the future, we will increase the number of subjects and further verify the proposed method by experiments. Also, we will consider including the Persistently Exciting of the matrix when estimating coefficients to generate the model. Furthermore, we will introduce a learning approach for updating sequential information in real time to convert estimated the EMG signal for the robot control signal.

## Figures and Tables

**Table 1:** Improvement of SN ratio by averaging method.

Number of Averaging	Improvement of SNR	
*M* (times)	$\sqrt{M}$ (times)	$10\log_{10}M$ (dB)
10	3.2	10.0
50	7.1	17.0
**60**	**7.7**	**17.8**
**70**	**8.4**	**18.4**
**80**	**8.9**	**19.0**
90	9.5	19.5
